# Toxicity of chlorhexidine on odontoblast-like cells

**DOI:** 10.1590/S1678-77572010000100010

**Published:** 2010

**Authors:** Fernanda Campos Rosetti LESSA, Andreza Maria Fabio ARANHA, Indri NOGUEIRA, Elisa Maria Aparecida GIRO, Josimeri HEBLING, Carlos Alberto de Souza COSTA

**Affiliations:** 1 Discipline of Pediatric Dentistry, Department of Orthodontics and Pediatric Dentistry, São Paulo State University, Araraquara Dental School, Araraquara, SP, Brazil.; 2 Discipline of Pathology, Department of Physiology and Pathology, São Paulo State University, Araraquara Dental School, Araraquara, SP, Brazil.

**Keywords:** Chlorhexidine, Odontoblasts, Cytotoxicity, Cell viability, Protein synthesis

## Abstract

Chlorhexidine gluconate (CHX) is recommended for a number of clinical procedures and it has been pointed out as a potential cavity cleanser to be applied before adhesive restoration of dental cavities. Objective: As CHX may diffuse through the dentinal tubules to reach a monolayer of odontoblasts that underlies the dentin substrate, this study evaluated the cytotoxic effects of different concentrations of CHX on cultured odontoblast-like cells (MDPC23). Material and Methods: Cells were cultured and exposed to CHX solutions at concentrations of 0.06%, 0.12%, 0.2%, 1% and 2%. Pure culture medium (α-MEM) and 3% hydrogen peroxide were used as negative and positive control, respectively. After exposing the cultured cells to the controls and CHX solutions for 60 s, 2 h or 60 s with a 24h recovery period, cell metabolism (MTT assay) and total protein concentration were evaluated. Cell morphology was assessed under scanning electron microscopy. CHX had a dose-dependent toxic effect on the MDPC-23 cells. Results: Statistically significant difference was observed when the cells were exposed to CHX in all periods (p<0.05). Significant difference was also determined for all CHX concentrations (p<0.05). The 60-s exposure time was the least cytotoxic (p<0.05), while exposure to CHX for 60 s with a 24-h recovery period was the most toxic to the cells (p<0.05). Conclusion: Regardless of the exposure time, all CHX concentrations had a high direct cytotoxic effect to cultured MDPC-23 cells.

## INTRODUCTION

With the remarkable development of resin materials and techniques that promote adhesion to dental structures, particularly the interaction of adhesive systems with dentin, different treatment of cavity walls with cleaning agents have been proposed^6^. The importance of using substances with antimicrobial properties for cleaning of cavity walls prior to application of adhesives systems has been emphasized. However, in addition to antimicrobial activity, a cavity cleanser should not interfere with the bonding mechanism during adhesive restoration, allowing complete diffusion of the bonding agent within the acid-etched dentin, and should inhibit or at least minimize the degradation of the adhesive interface by enzymatic components present in saliva and dentin structure, such as metalloproteinases (MMPs), maintaining the integrity of restoration over time^6,22^.

Odontoblasts are specialized cells that play a key role in the pulpal healing process and formation of the mineralized tissue barrier^1^. A chemical injury to the primary odontoblasts could impair the repair capacity of the pulpodentinal complex by inducing apoptosis or direct death of these cells due to a cytotoxic effect^6^. Therefore, in addition to the properties mentioned above, an ideal cavity cleanser should also present a low or preferably no toxic effects to pulp cells, especially odontoblasts^28^.

Chlorhexidine gluconate (CHX) is used in a number of dental procedures and has been pointed out as a potential cavity cleanser for cavities with or without pulp exposure. This antimicrobial agent possesses a broad spectrum of activity against a wide array of oral microorganisms, including Gram positive and Gram negative bacteria, bacterial spores, lipophilic viruses, yeasts and dermatophytes^8,9^. The optimal action of CHX solutions occurs within a specific pH range (5.5 to 7.0)^26^. In the same way as demonstrated for different chemical agents indicated for use as cavity cleansers or endodontic irrigants^6,17,24,26^, CHX also presents cytotoxic effects on different cell lines. In vitro experiments have been performed in an attempt to elucidate the mechanisms of action of CHX and have demonstrated its cytotoxic potential by inhibition of protein synthesis^14,25^, induction of apoptosis at low concentrations and necrosis at high concentrations^11^, in addition to inhibition of DNA synthesis^19^. The cytotoxic potential of CHX can also be related to the length of cell exposure^2^ and CHX concentration^27^. However, current investigation has demonstrated that CHX could be used as a cavity cleanser after caries removal because, in addition to its antimicrobial activity, it does not interfere with hybrid layer formation^3^ and inhibits the action of metalloproteinases^13^, delaying the degradation of the resin/dentin interface^18^.

Over the last decades, in vitro models that simulate the in vivo functioning of pulp cells have been developed to investigate the pulp response to different stimuli in a molecular level^15,20^. Studies using odontoblast-like cells are important because odontoblasts make up the layer of cells the line the periphery of the pulp and are the first cells affected by substances that reach the pulp chamber via transdentinal diffusion^7^. Therefore, in view of the current recommendation for clinical use of CHX as a cavity cleanser, it would be interesting to investigate the direct cytotoxic potential of this antimicrobial agent at concentrations similar to those of commercially available products on pulp cells. The purpose of this study was to evaluate the cytotoxicity of different concentrations of aqueous CHX solutions on cultured MDPC-23 cells after different exposure times.

## MATERIAL AND METHODS

Odontoblast-like cells (MDPC-23)^15^ were cultured in Minimum Essential Medium Eagle Alpha Modification (α-MEM; Sigma-Aldrich Corp., St. Louis, MO, USA) supplemented with 10% fetal calf serum (FCS; Gibco, Grand Island, NY, USA), with 100 IU/mL penicillin, 100 µg/mL streptomycin and 2 mmol/L glutamine (Gibco, Grand Island, NY, USA) in an humidified incubator (Isotemp Fisher Scientific, Pittsburgh, PA, USA) with 5% CO and 95% air at 37ºC. The cells were 2 sub-cultured at every 3 days at a concentration of 30,000 cells/cm^2^, until an adequate number of cells were obtained for the study.

### Analysis of Cell Metabolism

Cell metabolic activity was evaluated by succinic dehydrogenase (SDH) activity, which is a measure of the mitochondrial respiration of the cells. For such purpose, the methyltetrazolium (MTT) assay was used^23^.

A 20% CHX solution (Farmácia Escola, UNESP, Araraquara, SP, Brazil) was diluted in á-MEM culture medium to obtain the CHX concentrations evaluated in the study: 0.06, 0.12, 0.2, 1 and 2%. Negative and positive controls were pure culture medium (á-MEM) and 3% hydrogen peroxide (H_2_O_2_), respectively. The MDPC-23 cells were exposed to contact with the CHX solutions for different times: 60 s, 2 h and 60 s with a recovery period of 24 h. Ten samples per control and CHX solutions were used for analysis of cell metabolic activity and other 2 samples were processed for analysis of cell morphology under scanning electron microscopy (SEM).

MDPC-23 cells were seeded (30,000 cells/cm^2^) in 24-well plates (Costar Corp., Cambridge, MA, USA) and maintained in a humidified incubator with 5% CO_2_ and 95% air at 37ºC for 72 h. Thereafter, the culture medium was aspirated and the control and CHX solutions were added to each well containing the cells. After the pre-determined exposure times, the control and CHX solutions were aspirated and replaced by 900 µL of culture medium (α-MEM) and 100 µL of MTT solution (5 mg/mL phosphate buffered saline - PBS) in each well. The cells in contact with the MTT solution were incubated at 37ºC for 4 h. Thereafter, the solution was replaced by 600 µL of acidified isopropanol solution (0.04 N HCl). The absorbance was measured at 570 nm wavelength in a spectophotometer (ELX 800 - Universal Microplate Reader; Bio-Tek instrument, Inc., Winooski, VT, USA).

Three aliquots of each well (100 µL each) were transferred to a 96-well dish (Costar Corp., Cambridge, MA, USA). For standardization of absorbance reading, the first two wells were filled with 100 mL of the acidified isopropanol solution to determine the value corresponding to total passage of light, that is, the maximum value to reduce cell metabolism. The values obtained from the three aliquots were averaged to provide a single value. The final values obtained with the control and CHX solutions were submitted to statistical analysis by Mann-Whitney nonparametric test at 5% significance level.

### Analysis of Cell Morphology by Scanning Electron Microscopy

Two representative samples of each control and CHX solutions were submitted to analysis of cell morphology under SEM. For such purpose, 12-mm-diameter cover glasses (Fisher Scientific, Pittsburg, PA, US) were placed on the bottom of two wells before seeding of the MDPC-23 cells (30,000 cells/cm^2^). After the pre-determined exposure times, the control and CHX solutions were aspirated and the cells that remained adhered to the glass substrate were immersed in 1 mL of buffered 2.5% glutaraldehyde for 120 min. The cells were then submitted to 5 min rinses with 1 mL PBS (three times), post-fixed in 1% osmium tetroxide for 60 min and processed for examination by scanning electron microscope (DSM-940A, ZEISS, Oberkochen, Germany).

### Total Protein Concentration

Total protein concentration by Lowry method was performed in the 10 samples from the experimental and control groups. The culture medium was aspirated and the cells were washed three times with 2 mL PBS heated at 37ºC. Two milliliters of 0.1% sodium lauryl sulfate (Sigma-Aldrich Corp.) were added to each well and maintained for 30 min at room temperature to produce cell lysis. The samples were homogenized and 1 mL from each well was transferred to properly labeled Falcon tubes (Corning Incorporated, Corning, NY, USA). One milliliter of distilled water was added to the blank tube. Next, 1 mL of Lowry reagent solution (Sigma-Aldrich Corp.) was added to all tubes, which were agitated for 10 s in a tube agitator (Phoenix AP 56, Araraquara, SP, Brazil). After 20 min at room temperature, 500 µL of FolinCiocalteau’s phenol reagent solution (Sigma-Aldrich Corp.) were added to each tube followed by 10 s agitation. Thirty minutes later, three 100 µL aliquots of each tube were transferred to a 96-well dish and the absorbance of the test and blank tubes was measured at 620 nm wavelength using a spectrophotometer (ELX 800; Universal Microplate Reader). The absorbance values obtained in the tubes were transformed in total protein concentration by a standard curve.

### Statistical Analysis

As cell metabolism activity and total protein concentration data had a non-normal distribution, the Mann-Whitney non-parametric test was used for comparison of the groups and exposure times. Significance level was set at 5% (p < 0.05). The analysis of cell morphology was performed descriptively.

## RESULTS

### Cell Metabolism (MTT Assay)

The results of cell metabolism obtained after exposure of the MDPC-23 cells to the control and CHX solutions are presented in Table 1.

**Table 1:** Medians (P25-P75) of the absorbance values obtained in the cell metabolism (MTT) assay for the control and chlorexidine (CHX) solutions according to the exposure time

Groups*	**Exposure time**		
	**2 h**	**60 s**	**60 s +24-h recovery**
0.06 % CHX	0.1947 (0.1863-0.2103) A,a**	0.2679 (0.2370-0.2815) AB,b	0.1200 (0.1166-0.1300) A,c
0.12% CHX	0.1679 (0.1601- 0.1736) B,a	0.2591 (0.2457-0.2676) A,b	0.1239 (0.1140-0.1294) A,c
0.2% CHX	0.1535 (0.1472-0.1544) C,a	0.2359 (0.2009-0.2964) ABC,b	0.1174 (0.1121-0.1275) AB,c
1% CHX	0.1408 (0.1373-0.1442) D,a	0.2437 (0.2277-0.2552) B,b	0.1184 (0.1094-0.1247) AC,c
2% CHX	0.1264 (0.1226-0.1381) E,a	0.2123 (0.1941-0.2211)C,b	0.1131 (0.1062-0.1177) BC,c
α-MEM	0.5661 (0.5438-0.5961) F,a	0.4616 (0.3811-0.4691) D,b	0.4902 (0.4732-0.5357) D,c
H_2_O_2_	0.0700 (0.0666-0.0725) G,a	0.1211 (0.1121-0.1327) E,b	0.1291 (0.1176-0.1363) A,b

* n=10 for each period within the same group;

** Different uppercase letters in columns and different lowercase letters in rows indicate statistically significant difference (Mann-Whitney. p>0.05).

There was statistically significant difference (p<0.05) among the control and CHX solutions as well as among the exposure times. All CHX concentrations caused an intense toxic effect to the MDPC-23 cells. CHX concentrations of 0.06% and 0.12% caused less toxic effects to the cells and were not significantly different from each other (p>0.05). Higher cytotoxicity to the MDPC23 cells was observed as the CHX concentration increased, characterizing a dose-dependent toxic effect of this chemical agent. The positive control (3% H_2_O_2_) was the most cytotoxic to the cultured MDPC-23 cells. Overall, CHX concentrations of 0.06%, 0.12%, 0.2%, 1.0% and 2.0% decreased cell metabolism by 61%, 63%, 65%, 67% and 70%, respectively.

There was statistically significant difference (p<0.05) among all CHX concentrations for all exposure times. The 60-s exposure time was the least cytotoxic (p<0.05), while exposure to CHX solutions for 60 s with a 24-h recovery period was the most toxic to the cells (p<0.05).

### Cell Morphology (SEM)

**Figure 1:**
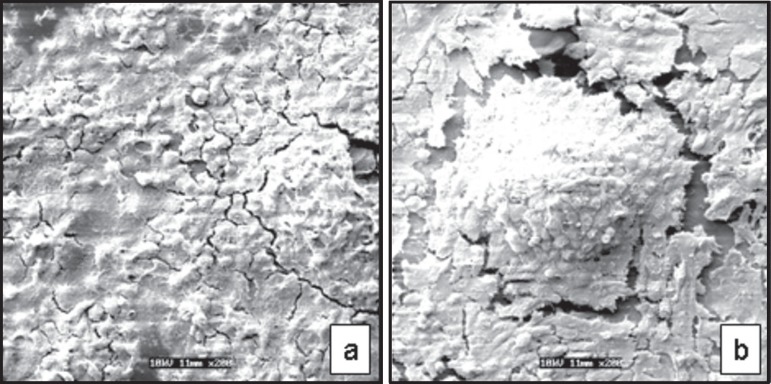
Negative control (α-MEM). Scanning Electron Microscopy original magnification ×200. (a): MDPC-23 cells adhered to the glass substrate. near confluence. (b): Cells organized as epithelioid nodules

Two samples representative of the control and CHX solutions were selected for analysis of the morphology of the MDPC-23 cells that remained adhered to the glass substrate. In the negative control group (α-MEM), in all exposure times, the MDPC-23 cells were near confluence and were organized as epithelioid nodules (Figure 1a/b).

**Figure 2:**
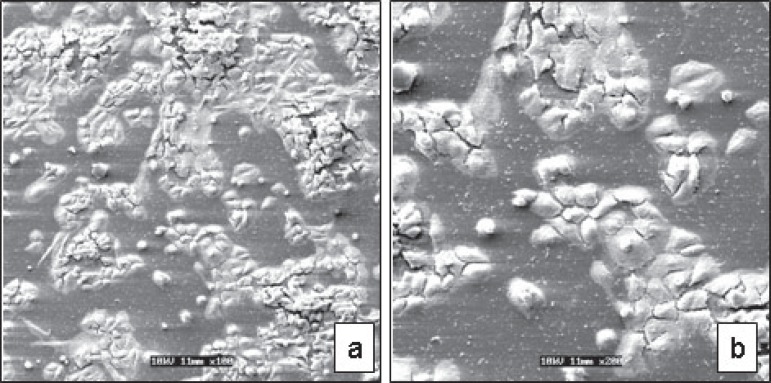
0.06% chlorexidine - 60 s. (a): A marked alteration of cell morphology was observed for all exposure times [Scanning Electron Microscopy (SEM) original magnification ×100)]. (b): Detail of Fig. 3a at greater magnification showing a smaller number of cells adhered to the glass substrate. (SEM original magnification ×200)

**Figure 3:**
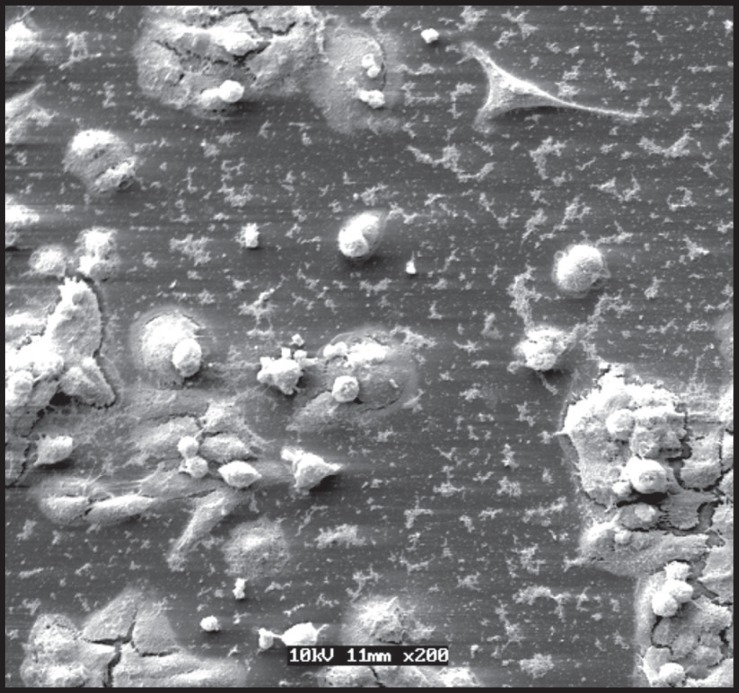
1 % chlorexidine - 60 s +24-h recovery: Note the smaller number of remaining cells and rests of cytoplasmatic processes that detached from the substrate (Scanning Electron Microscopy original magnification ×200)

A marked alteration of cell morphology and a small number of cells adhered to the glass substrate were observed for all exposure times (Figure 2a/b). These events were more accentuated as the CHX concentration and the contact time with the cells increased. A larger number of cells remained adhered to the glass substrate when the CHX solution was applied to the cells for 60 s (Figure 2a/b). Therefore, for the lowest CHX concentrations and shortest exposure times, cells with similar morphology to those of the negative control group were observed, though in a smaller number. On the other hand, the number of MDPC-23 cells that remained adhered to the glass substrate decreased progressively as CHX concentration increased. These cells presented a smaller size and round shape (Figure 3). Extensive cell-free areas and a large amount of membrane cell debris were also found.

**Figure 4:**
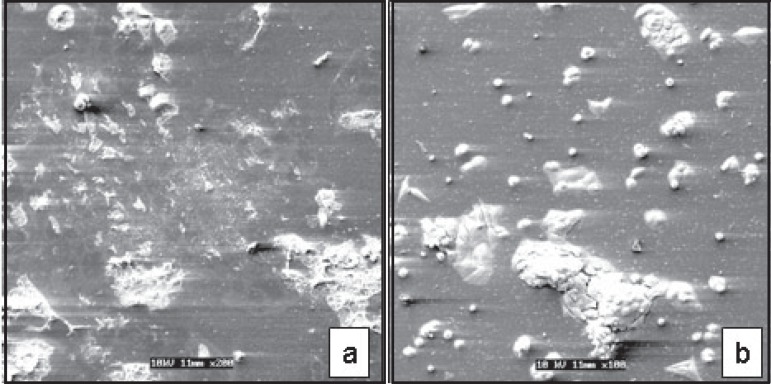
(a): Positive control (3% H2O2) - 2h. Some of the MDPC-23 cells remained adhered to the glass substrate presented a round shape and total or partial loss of cytoplasmatic processes [Scanning Electron Microscopy (SEM) original magnification ×200)]. (b): 2% chlorexidine (CHX) - 2h. Similar morphology as positive control cells was observed when the cells were treated with 2% chlorexidine (CHX). (SEM original magnification ×100)

In the positive control group (3% H2O2), the small number of MDPC-23 cells that remained adhered to the glass substrate presented a round shape as well as total loss or maintenance of few cellular processes on the cytoplasmic membrane (Figure 4a). These morphological characteristics of the few cells adhered to the glass substrate were also observed when MDPC-23 cells were exposed to 2% CHX (Figure 4b).

### Total Protein Concentration (Lowry Method)

The results of total protein concentration obtained after exposure of the MDPC-23 cells to the different control and CHX solutions are presented in Table 2.

**Table 2:** Medians (P25-P75) of the total protein values (µg/mL) obtained for the control and chlorexidine (CHX) solutions according to the exposure time

Groups*	**Exposure time**		
	**2 h**	**60 s**	**60 s +24-h recovery**
0.06% CHX	111.62 (101.61-120.02) A,a*	120.83 (114.87-127.33) A,b	95.92 (92.94-100.52) A,c
0.12% CHX	98.09 (95.37-110.27) AB,a	115.96 (110.81-121.37) AB,b	94.83 (87.52-96.73) A,c
0.2% CHX	97.55 (93.21-101.34) B,a	112.71 (107.02-118.39) BC,b	88.88 (84.82-92.12) B,c
1% CHX	88.34 (83.73-90.23) C,a	107.84 (105.12-111.89) C,b	76.96 (72.36-80.21) C,c
2% CHX	81.84 (80.21-85.90) C,a	102.96 (100.79-104.85) D,b	70.46 (67.21-72.36) D,c
α-MEM	179.87 (163.07-220.49) D,a	138.16 (136.54-148.99) E,b	159.83 (157.39-164.97) E,c
H_2_O_2_	71.55 (68.30-76.15) E,a	76.96 (71.55-81.29) F,b	70.46 (69.11-73.17) D,a

* n=10 for each period within the same group;

** Different uppercase letters in columns and different lowercase letters in rows indicate statistically significant difference (Mann-Whitney. p>0.05).

There was statistically significant difference (p<0.05) among the control and CHX solutions as well as among the exposure times. The cells exposed to the CHX for only 60 s presented greater total protein concentration followed by 2 h and 60 s exposure with 24-h recovery. Regarding CHX concentrations, the reduction of total protein concentration occurred in a dosedependent manner.

## DISCUSSION

Due to its recognized antimicrobial effect and other beneficial properties, CHX has been subject of investigation in different biomedical areas. Despite the several positive properties of CHX, which include non-interference with the adhesion between the bonding agent and the dentin substrate^8^ and inhibition of dentin metalloproteinases^30^, a previous in vitro study has demonstrated its toxic effect on eukaryotic cells associated to decrease of protein synthesis^25^. CHX may also interfere with the mitochondrial respiration of cells^4^, inhibiting DNA synthesis and cell proliferation^19^. Nevertheless, the specific mechanisms of action of CHX on the cells have not yet been fully elucidated. In the present study, an in vitro experiment was performed to evaluate the toxicity induced by CHX at different concentrations and determine whether the cytotoxic effects of this chemical agent on MDPC23 cells are related to length of its contact with the cells. All CHX concentrations were more toxic to the MDPC-23 cells after a 2-h exposure time compared to an exposure of 60 s. This result indicates that, regardless of the concentration, the longer the contact time of the cells with CHX, the more intense the cytotoxic effect of this chemical agent. However, the most intense CHXinduced cytotoxicity occurred when the cells were exposed to the different CHX concentrations for 60 s and allowed to recover for 24 h. This result indicate that even after being removed from the direct contact with the cultured cells, CHX maintains its action over time, interacting with the cell structures, either causing direct damage or inhibiting their metabolism. This continuous effect of CHX on the cells is due to the acknowledged substantivity of this antimicrobial agent^10,29^. The lack of recovery of cultured cells after contact with CHX has been demonstrated in a previous study^25^, in which human fibroblasts were exposed to 0.12% CHX for 30 s and incubated for recovery period of 7 days. The authors found by analysis of cell proliferation and viability that the cells did not recover within the established period. Similar results were found by Mariotti and Rumpf^21^ (1999), who demonstrated that exposure of human fibroblasts to 0.12% CHX for 1, 5 and 15 min with a 24-h recovery period reduced the proliferation of cells by 72.7%. Cell proliferation was dependent on CHX concentration in cell culture but independent of the duration of CHX exposure. The reduction in cell metabolism observed in the present study, especially for the higher CHX concentrations, may be due to the inhibition of mitochondrial activity of the cells or intense direct cell death, as observed in the SEM analysis of cell morphology and number of cells that remained adhered to the glass substrate. Therefore, it seems liable to assume that the use of CHX in cavities with pulp exposure should not be recommended because this chemical agent maintains its cytotoxic effects to the pulp cells even after being rinsed off tooth surface. Regarding the use of CHX as a cavity cleanser in teeth without pulp exposure, further research should be performed to evaluate the capacity of diffusion of this substance through different dentin thicknesses as well the relationship between the concentration of CHX applied to the dentin cavity floor and the one that could reach the pulp space.

Over the last decades, a wide array of cell lines has been used to evaluate cytotoxicity of CHX. Hidalgo and Dominguez^19^ (2001) have demonstrated that exposure of cultured human dermal fibroblasts to CHX at concentrations equal to or greater than 0.005% for 3 h caused cell death. Goldschmidt, et al.^14^ (1977), on the other hand, evaluated the exposure of cultured human fibroblasts to CHX at similar concentrations and for the same contact time, though using a different evaluation technique, and did not observed cell death. A recent study has demonstrated that exposure of L929 fibroblasts to a CHX concentration as low as 0.016% for 24 h increased the necrosis rate of these cells in 79.77%^11^. Chang, et al.^5^ (2001) have reported that exposure of human periodontal ligament fibroblasts to 0.125% CHX for 120 s caused almost complete inhibition of the mitochondrial activity of these cells. The methodological variations observed in the studies that investigated the effects CHX solutions on cell cultures may explain the diversity of results found in the literature. In the present study, the cytotoxicity of CHX was evaluated on MDPC-23 cells because in mammalian teeth the odontoblasts are organized in a monolayer that underlies the coronal and root dentin^1^. Therefore, any material that is capable to diffuse through the dentinal tubules will first interact with these peripheral pulp cells, which play an important role in pulp healing^16^. As the application of 2% CHX on the cavity walls after caries removal has been recommended in the literature^4,18^, the present study, as a first investigation, intended to demonstrate which CHX concentration would cause pulp cell damage. It is known that dentin acts as a true biological barrier, providing protection to the pulp cells^12^. Therefore, it is expected that CHX at a low concentration could reach the pulp space after application of this substance as a cavity cleanser in the same way as the 2% CHX. CHX concentrations ranging from 0.06% to 2% were evaluated in the present study. It was observed that all CHX concentrations were toxic to the MDPC-23 cells in a dosedependent manner. The percentage of inhibition of cell metabolism for CHX concentrations of 0.06 to 2% ranged from 42% and 78%, respectively. It should be emphasized that in the present study FCS was not added to the culture medium during dilution of CHX to obtain the final concentrations used in the experiment because it has been demonstrated^21^ that supplementation of the culture medium with FCS at concentrations from 0.1 to 10% caused immediate precipitation of CHX. This finding was confirmed by Hidalgo and Dominguez^19^ (2001) , who verified that 10% FCS added to the culture media appeared to have an attenuating effect against CHX-induced cytotoxicity, permitting higher cell survival, ATP intracellular levels and DNA synthesis. This occurred presumably due to the non-specific binding of CHX to serum proteins, leading to a lower availability of the drug to act on the cultured cells. According to some authors, one of the mechanisms of action of CHX on cultured cells is the inhibition of protein synthesis. Pucher and Daniel^25^ have demonstrated that a 30-s application of 0.12% CHX on cultured cells reduced total protein synthesis by approximately 50%, while Mariotti and Rumpf^21^ (1999) reported that gingival fibroblasts exposed to 0.12% CHX for 1 min followed by a 24-h recovery period had a 98.8% and 98.2% reduction in collagen and noncollagen protein production, respectively. Goldschmidt, et al.^14^ (1977) also demonstrated that protein production was inhibited by 97% after exposure of a fibroblast culture to 0.2% CHX for 3 h. In the present study, inhibition of total protein synthesis ranged from 12% to 56% depending on the CHX concentration to which the MDPC-23 cells were exposed. This finding demonstrates that CHX-induced inhibitory activity of protein synthesis was also dose-dependent. Unlike Mariotti and Rumpf^21^ (1999), who found that even CHX concentrations with little effect on cellular proliferation reduced significantly both collagen and noncollagen protein production of human gingival fibroblasts, the results of the present study showed that the decrease in protein synthesis by the MDPC-23 cells exposed to CHX accompanied the reduction of cell metabolism.

Regarding cell morphology, more significant alterations were observed as the concentration of the CHX solutions increased. Also, the longer the exposure time to the CHX solutions, the more accentuated the morphological alterations of the MDPC-23 cells. The cells were small-sized and had a round shape. Large cell-free areas or areas presenting remains of the disrupted cell membrane were found on SEM analysis. These findings indicate a direct correlation between CHX concentration and its toxic effects to MDPC-23 cells. Similar results have been reported by Souza, et al.^27^ (2007), though using lower CHX concentrations.

The findings of the present study clearly demonstrated the cytotoxic effects of aqueous CHX solutions at different concentrations applied for different times on cultured MDPC-23 cells. However, it should be emphasized that the results of this in vitro cytotoxicity assay have limitations for a direct extrapolation to clinical conditions, especially when the dentin is interposed between the chemical agent and the pulp cells. Further research should be conducted to investigate the possible transdentinal diffusion of CHX solutions applied on different thicknesses of dentin discs and the effects of their extracts on odontoblast cell lines. These studies will substantiate a safer and more effective clinical use of CHX solutions as cavity cleansers.

## CONCLUSION

Under the tested conditions it may be concluded that all aqueous CHX solutions applied for different times on cultured MDPC-23 cells presented a dose- and time-dependent cytotoxicity. The higher the CHX concentration and the longer the contact time with the cells, the stronger its cytotoxic effects. The MDPC-23 cells did not recover from the immediate CHXinduced cytotoxic effects.
